# Anuria Secondary to Bilateral Obstructing Ureteral Stones in the Absence of Renal Colic

**DOI:** 10.1089/cren.2016.0055

**Published:** 2016-05-01

**Authors:** Carolyn A. Salter, Christopher Lang, Hernan O. Altamar

**Affiliations:** ^1^ Department of Urology, Walter Reed National Military Medical Center at Bethesda, Bethesda, Maryland.; ^2^ Department of Emergency Medicine, Walter Reed National Military Medical Center at Bethesda, Bethesda, Maryland.

## Abstract

***Background:*** Obstructing ureteral stones are a rare cause of anuria, which is typically from prerenal or renal etiologies. Classically, obstructive stones cause moderate to severe renal colic. Urolithiasis is rarely considered during evaluation of painless anuria.

***Case Presentation:*** We present an unusual case of a 73-year-old Caucasian female who presented with anuria and was found to have large bilateral obstructing ureteral stones in the absence of renal colic.

***Conclusion:*** Given that patients with obstructive anuria can be asymptomatic, urolithiasis should be considered in all patients presenting with anuria.

## Introduction and Background

Nephrolithiasis is occurring with increased incidence in the United States with a prevalence of 13% currently, up from 5.25% in the 1990s and markedly increased from 3.8% in the 1970s. Worldwide, prevalence is higher in the Middle East with rates of 20% in Saudi Arabia.^1^ The majority of patients with ureteral stones present with moderate or severe renal colic, with proximal ureteral stones causing flank or upper abdominal pain and more distal stones causing pain in the groin, scrotum, or labia.^1^ They tend to have normal urine output during the episode.

## Presentation of Case

The patient is a 73-year-old Caucasian female who presented to the emergency department with the chief complaint of anuria for almost 24 hours. She also complained of malaise, anorexia, nausea, and subjective fever and chills. When queried, she endorsed recent gross hematuria, which had resolved before her anuria. The patient denied any flank or abdominal pain consistent with renal colic.

She was afebrile with normal vital signs. A bedside bladder scan revealed minimal urine volume of 30 mL. The patient had no abdominal pain or costovertebral angle tenderness on examination. Her laboratories were notable for leukocytosis to 20,500/mcL (20.5 × 10^9^/L) and acute kidney injury (AKI) with blood urea nitrogen (BUN) and creatinine of 54.0 mg/dL (19.28 mmol/L) and 7.11 mg/dL (628.52 μmol/L), respectively, up from a recent baseline BUN and creatinine of 17.0 mg/dL (6.07 mmol/L) and 0.8 mg/dL (70.72 μmol/L), respectively. She was able to produce a scant urine sample that revealed 17 red blood cells, 13 white blood cells, and proteinuria. Urine culture was subsequently negative. Her medical history was notable for prior kidney stones with three episodes of spontaneous stone passage. She had presented with pain typical of renal colic during these previous stone episodes. The patient had never been evaluated by urology for her nephrolithiasis. In terms of her medical problems, she had diabetes, hypertension, and breast cancer. Her diabetes was controlled with metformin and recent HbA1c before this episode was 6.7. The patient did have mild peripheral neuropathy in her bilateral feet as determined through a monofilament test performed by her primary care provider during a routine physical examination. Due to her anuria, acute renal failure, and prior nephrolithiasis, there was concern for obstructing ureteral stones as the etiology.

**FIG. 1.**
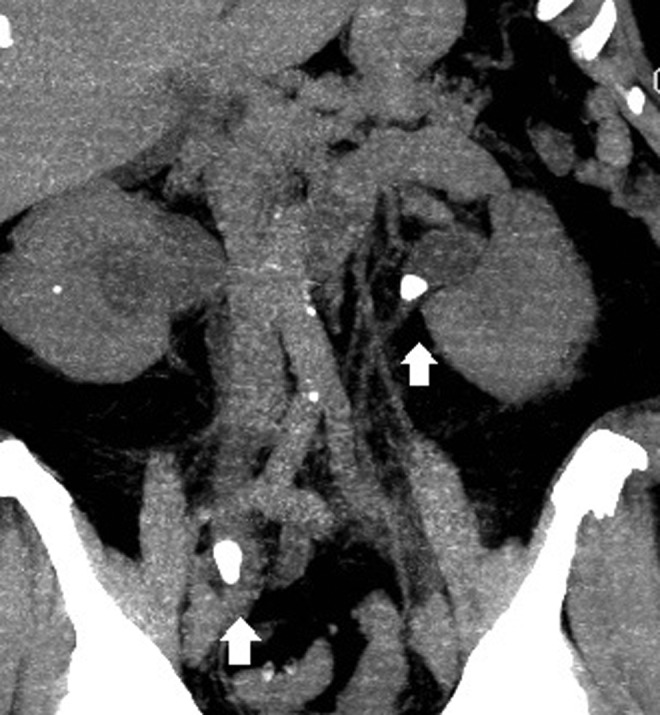
Noncontrast CT scan with 1.5 cm stone in right distal ureter and 1.1 cm stone at left ureteropelvic junction with associated hydronephrosis.

A noncontrast CT scan was performed that revealed a 1.5 cm stone in the right distal ureter and a 1.1 cm stone at the left ureteropelvic junction with associated severe and moderate hydronephrosis (Fig. 1). Urology was consulted for bilateral obstructing ureteral stones. The patient was taken from the emergency department to the operating room that evening for cystoscopy with bilateral ureteral stent placement. She recovered in the intensive care unit postoperatively for close monitoring of her electrolyte and fluid balance because of concern for postobstructive diuresis. She had an uneventful hospital course and her urine output returned back to baseline after significant diuresis with over 6 L/day in the immediate postoperative period. The patient was discharged home on postoperative day 3 with a markedly improved renal function with a creatinine of 1.1 mg/dL (97.24 μmol/L). She had further normalization of her renal function as an outpatient and subsequently underwent bilateral ureteroscopy with laser lithotripsy for her ureteral stones. Intraoperatively, no ureteral strictures were seen. The bilateral ureteral stones were fragmented but a right lower pole stone was left *in situ*. Her stones seemed consistent with uric acid composition based on their appearance, radiolucency on fluoroscopy, and density on CT. She was started on potassium citrate postoperatively. Subsequent stone analysis confirmed uric acid stones with composition 83% urate, 10% calcium oxalate monohydrate, 5% calcium oxalate dehydrate, and 2% calcium phosphate. The patient is currently asymptomatic with normal renal function. She has opted against further metabolic evaluation or additional surgery for her right lower pole stone.

## Discussion and Literature Review

AKI as defined by the RIFLE criteria has three severity classes: risk, injury, and failure, with each class having criteria based on glomerular filtration rate (GFR) and urine output. Risk is defined as 1.5× increase in baseline creatinine or 25% decrease in GFR with urine output of <0.5 mL/kg/hr for 6 hours. Injury is more severe with a twofold increase in creatinine or at least a 50% decrease in GFR and urine output of <0.5 mL/kg/hr for 12 hours. The most severe RIFLE stage is failure, which is defined as 3× increase in creatinine or decline in GFR of at least 75% or creatinine of more than 4 mg/dL (353.60 μmol/L). The urine output criterion for this classification is anuria for 12 hours or urine output of <0.3 mL/kg/hr for 24 hours.^2^ Based on this classification system, our patient was in the most severe stage of AKI.

Obstructing ureteral stones are an unusual source of AKI, as postrenal causes overall encompass only 10%–12% of AKI cases. Within this group of postrenal AKI, nephrolithiasis is a rare etiology and causes an estimated 1%–2% of all events.^3^ There are limited data explicitly addressing the prevalence of anuria secondary to nephrolithiasis because of the rarity of this condition. One study that did address this issue evaluated patients with acute renal failure secondary to obstructing urolithiasis and found that only 11.1% of these patients had anuria.^4^

This case is even more unusual in that the patient had no classic symptoms of nephrolithiasis. Her chief complaint was anuria and she denied flank or abdominal pain. This is in contrast to more typical cases who usually present with moderate to severe renal colic.^1^ Our patient did have known diabetes and although it was controlled with metformin and she only had mild peripheral neuropathy, her diabetes could have contributed to her absence of symptoms. Providers should have a high index of suspicion to swiftly diagnose obstructing ureteral stones in patients with atypical presentations. Studies have shown that timely relief of obstruction is needed and that recovery of renal function depends on the degree and duration of the obstruction.^3^ With prompt diagnosis in the emergency department, the patient was able to undergo rapid surgical decompression and had almost complete normalization of her renal function before hospital discharge.

## Conclusion

Although obstructing ureteral stones are not typically considered as an etiology for painless anuria, this case shows that patients with this disease may be asymptomatic. Thus, obstructing ureteral stones should be included in the differential diagnosis of anuric patients as prompt diagnosis and treatment can lead to more marked improvements in renal function.

## Abbreviations Used

AKI: acute kidney injury
BUN: blood urea nitrogen
CT: computed tomography
GFR: glomerular filtration rate

## References

[1] Brener ZZ, Winchester JF, Salman H, Bergman M Nephrolithiasis: Evaluation and management. South Med J 2011;104:133–139 21258231 10.1097/SMJ.0b013e318206f6bd

[2] Kellum JA Acute kidney injury. Crit Care Med 2008;36(4 Suppl):S141–S145 18382185 10.1097/CCM.0b013e318168c4a4

[3] Tang X, Lieske JC Acute and chronic kidney injury in nephrolithiasis. Curr Opin Nephrol Hypertens 2014;23:385–390 24848936 10.1097/01.mnh.0000447017.28852.52 PMC4096690

[4] Jiang H, Wu Z, Ding Q Ureteroscopy and holmium: YAG laser lithotripsy as emergency treatment for acute renal failure caused by impacted ureteral calculi. Urol 2008;72:504–507 18653216 10.1016/j.urology.2008.05.041

